# Ethical

**Published:** 1889-10-19

**Authors:** 


### ETHICAL.

H. H., L.R.C.P., and L.S.A. writes: "I would feel deeply obliged by your kindly answering the following questions in your next issue. A. possesses M.R.C.S. only; B. an L.R.C.P., and L.S.A., engages as assistant, and signs an agreement, in which he is described as a surgeon, not to oppose A. As A. does not hold a medical qualification, and cannot recover in a county court for medical attendance, does the agreement hold good? That is, can A. legally restrain B. from opposing him in the future?"

⁂ The agreement not to practice, it is assumed, was absolute, and not contingent in any way upon the nature of the qualifications possessed by A. or B. If it was absolute, and not contingent, A. can certainly restrain B. from practising. Moreover, B., as an honourable man, ought to respect the spirit of the agreement, and should on no account think of practising after signing a bond to the contrary.

